# UVC-LED-based face mask design and efficacy against common germs

**DOI:** 10.2478/aiht-2023-74-3766

**Published:** 2023-12-29

**Authors:** Ali Gelir, Faruk Asicioglu, Aysegul S. Yilmaz, Mert Kuskucu, Mehmet Doymaz, Onur B. Özdemir, Devrim Sarıbal, Seda Salman, Ömer F. Kadi, Sedat Özdemir, Sinem N. Seyhan

**Affiliations:** Istanbul Technical University, Physics Engineering Department, Istanbul, Turkey; Istanbul University-Cerrahpaşa, Institute of Forensic Science and Legal Medicine, Istanbul, Turkey; Istanbul University-Cerrahpaşa Faculty of Medicine, Istanbul, Turkey; BezmialemVakıf University, Department of Medical Microbiology, Istanbul, Turkey; Haliç University Faculty of Medicine, Istanbul, Turkey

**Keywords:** antibacterial mask, A/Puerto Rico/8/1934 influenza virus, corona virus, COVID-19, *P. aeruginosa*, protective mask, public health, *S. aureus*, antibakterijska maska, COVID-19, javno zdravstvo, koronavirus, virus influence A/Puerto Rico/8/1934, *P. aeruginosa*, zaštitna maska, *S. aureus*

## Abstract

During the Covid-19 pandemic, one of the best means of personal protection was using face masks. In this context, the World Health Organization has declared the attempts to produce masks inactivating airborne virus species a welcome initiative. This preliminary study aimed to prove that airborne germs passing through a mask filter cartridge can be destroyed by the rays emitted from UVC LEDs placed in such cartridge. We therefore designed such a face mask and tested the efficiency of UVC LEDs placed in its cartridge against common contaminants, gram-positive *Staphylococcus aureus*, gram-negative *Pseudomonas aeruginosa*, and the influenza A/Puerto Rico/8/1934 virus because of its similarity with SARS CoV-2. Eight UVC LEDs with a total power of 75 mW provided sufficient germicidal effect for all three germs. In terms of safety, ozone production released during UVC LED emission was negligible. Our findings are promising, as they show that well-designed UVC-based face masks can be effective against airborne germs, but further research on a greater sample may help us learn more and optimise such face masks.

## Abstract (sl)

Tijekom pandemije Covida-19 jedan od najboljih oblika osobne zaštite bilo je nošenje maski za lice. U tom je smislu Svjetska zdravstvena organizacija pozdravila pokušaje izrade maski koje ubijaju virusne vrste koje se prenose zrakom. Cilj je ovoga preliminarnog istraživanja bio dokazati da se zrakom nošene klice koje prolaze kroz filtarske uloške mogu uništiti zračenjem UVC ledica smještenih u takve uloške. Stoga smo osmislili masku za lice s tom namjenom i iskušali djelotvornost UVC ledica protiv uobičajenih izvora zaraza: gram-pozitivnoga *Staphylococcus aureus*, gram-negativnoga *Pseudomonas aeruginosa* i virusa influence A/Puerto Rico/8/1934 zbog njegove sličnosti s virusom SARS CoV-2. Osam UVC ledica ukupne snage 75 mW iskazale su dovoljan germicidni učinak protiv svih triju klica. U smislu sigurnosti primjene, ozon proizveden tijekom UVC-LED zračenja pokazao se zanemarivim. Naši su rezultati obećavajući jer pokazuju da dobro osmišljene maske za lice s UVC zračenjem mogu biti djelotvorne protiv zrakom nošenih klica, ali će tek daljnja istraživanja na većem uzorku pomoći da doznamo više i usavršimo takve maske za lice.

The recent COVID-19 pandemic has shown that air transmission of viruses on droplets can be devastating (1) but also that the use of face masks is quite effective against their spread. The World Health Organization (WHO) has therefore welcomed the initiative to design and produce masks inactivating airborne virus species transmitted by droplets (2).

In this context, UVC light has been known to possess germicidal properties since the late 1800s (3, 4), and there were many attempts to use it as part of upper-room ultraviolet germicidal irradiation (UVGI) technology to control the spread of measles (5), tuberculosis (6), HIV (7), and Sars-Cov2 (8, 9). Today, UV light has been widely used in healthcare facilities, food-processing plants, schools, and laboratories, as it effectively purifies air from bacteria, moulds, viruses, and fungi, especially in the 220–280 nm wavelength range (10, 11).

Although UV face masks have been in use since 2007 (12), the global COVID-19 pandemic has made them more popular, and a number of companies and organisations have designed their own versions. One of the versions uses LEDs with wavelengths between 200 and 300 nm and consists of a tiny anti-microbial polymer cover secured with straps like a regular face mask (13) Another uses high-intensity (25 mW/cm^2^) UVC light to disintegrate the genetic material of microorganisms and promises six hours of battery life. One uses a small external irradiation chamber attached to a belt connected via a flexible tube with a battery life of up to four hours (14) One mask has a ZnO/TiO_2_ photocatalytic bilayer combined with a UVC-LED layer as additional protection to use as needed (15).

Our design, in turn, uses a cartridge containing UVC LED lamps. The aim of this preliminary study was to see how effective this face mask and the cartridges would be against two most common causes of hospital infections, the gram-positive *Staphylococcus aureus* and gram-negative *Pseudomonas aeruginosa*, and an enveloped RNA influenza virus A/Puerto Rico/8/1934 because of its similarity with the severe acute respiratory syndrome coronavirus 2 (SARS CoV-2) responsible for the COVID-19 pandemic. Our hypothesis was that the emitted UVC LED light placed in the cartridges would suffice to destroy these three pathogens.

## MATERIALS AND METHODS

### Prototype face mask design

**Figure 1:**
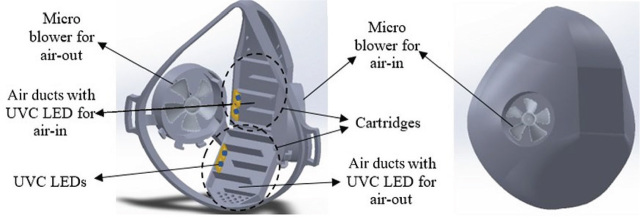
The skeletal (left) and final shape (right) of the face mask we designed. The schematic on the left shows blower slots and the cartridges with air ducts and UVC LEDs

**Figure 2:**
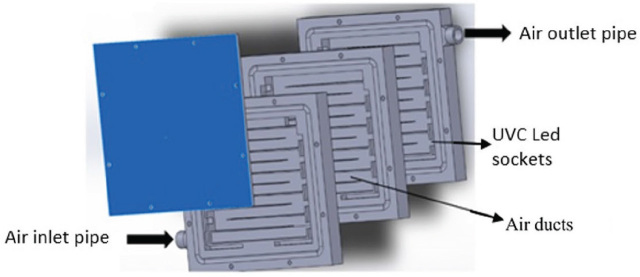
The internal structure of the three-layer cartridge (100×100×10 mm) used in the experiment

**Figure 3:**
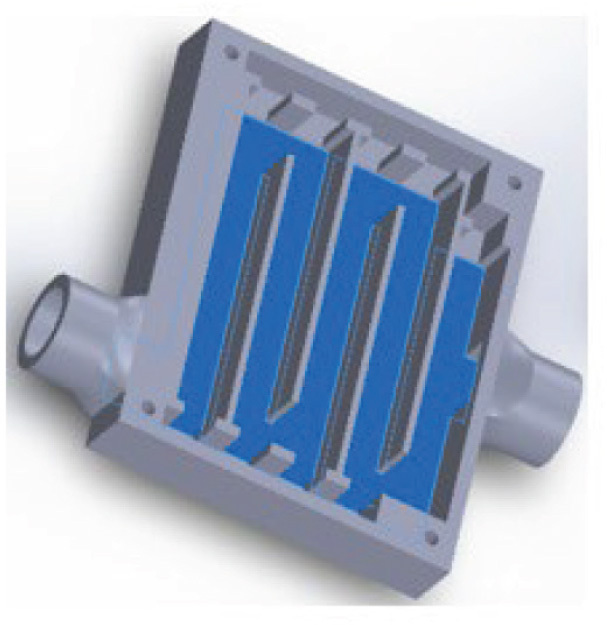
The internal structure of the one-layer cartridge (50×50×6 mm) used in the experiment

Figures 1–3 show the design of the face mask with detailed depiction of the UVC LED cartridges. The air is inhaled and exhaled through inlet and outlet air ducts in the cartridges whose air flow is adjusted with micro blowers to 5–10 L/min by changing the rotation speed.

Both inhaled and exhaled air are exposed to UVC light during the flow. The mask and the cartridges were designed with a Solidworks 3D CAD program (Dassault Systèmes SolidWorks Corporation, Waltham, MA, USA) and printed with a Flashforge Creator 3 3D printer (Zhejiang Flashforge 3D Technology Co., Ltd., Zehjiang, China) using the acrylonitrile-butadiene-styrene (ABS) filament for high durability.

### Determination of optimum UVC LED power

For this study we designed two different cartridges. The first is a three-layer cartridge with a total of 30 UVC LEDs and the output of about 150 mW (30×5 mW/LED). To reduce the mask size, we then determined the minimum number of LEDs required for acceptable disinfection (Figure 2) by starting with all 30 UVC LEDs turned on and taking measurements, and then proceeding by turning off the LEDs one by one to eventually conclude that eight provided optimum efficiency. The second cartridge consists of only one layer with eight UVC LEDs (Figure 3), which we used for this study.

The UVC-LEDs we used are the Lekoled 3535 model (ShenZen, China) with a 275 nm emission wavelength and average radiant flux of 10 mW. They were powered by a battery (6.7 V, 80 mA for the three-layer cartridge and 6.7 V, 200 mA for the one-layer cartridge) and connected with a general-purpose LED driver circuit based on adjustable DC-DC converter with adjustable current designed in our laboratory.

### Microbiological testing

**Figure 4:**
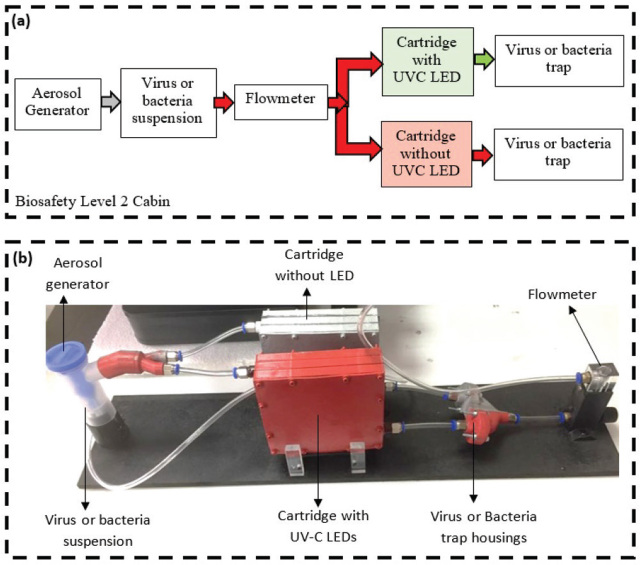
Experimental setup with active and control cartridges

Figure 4 shows the experimental setup for bacterial and viral determination. To generate aerosol, we used a general-purpose nebuliser (Nimomed, Denizli, Turkey) with a capacity to produce 15 L/min of aerosol of around 5 μm particle size. Air flow through the cartridge was adjusted to around 6 L/min to simulate normal breathing.

At the cartridge outlet the bacteria were trapped on the Whatman filter paper, and the viruses in cold serum-free Dulbecco's Modified Eagle's Medium (DMEM) (Merck, Darmstadt, Germany) at the bottom of Falcon tubes. Fresh cultures of the *Staphylococcus aureus* and *Pseudomonas aeruginosa* strains were taken from the Istanbul University-Cerrahpaşa Faculty of Medicine Infection Control Laboratory and COVID-19 Diagnostic Laboratory and serially passaged in Mueller Hinton agar as described elsewhere (16). A suspension containing 10^6^ CFU/mL of bacteria was cultivated in a sterile saline solution and this suspension placed in the nebuliser chamber.

To determine bacterial contamination, we removed the filters located at the cartridge output and transferred to 50 mL Falcon tubes containing 5 mL of sterile saline solution. After two minutes of vortexing, we took 100 mL of liquid from the tubes and inoculated blood agar (Laborlar Biotecnology, Istanbul, Turkey), CHROMagar (Laboratorios Conda S.A., Madrid Spain), and MacConkey agar media (Biolab Diagnostics Laboratory Inc., Budapest, Hungary). The combined use of these three media provides a versatile toolset for the identification, isolation, and analysis of a wide range of microorganisms (17, 18). Blood agar supports the growth of challenging microorganisms and is used for assessing haemolysis reactions in standard *in vitro* microbiological analyses (excluding living cells). CHROMagar enables fast and reliable identification of various pathogenic microorganisms based on their colour. MacConkey agar is used to isolate gram-negative enteric bacteria and distinguish those lactose-fermenting from nonlactose-fermenting. After 24 h, we counted the colonies.

Influenza A/Puerto Rico/8/1934 (PR8) strain was propagated in Madin Darby Canine Kidney (PMID: 34960614) (MDCK) (Biosys, Karben, Germany) cells grown at 37 °C in 5 % CO_2_ Dulbecco's Modified Eagle's Medium (DMEM) (Merck, Darmstadt, Germany) supplemented with 10 % foetal bovine serum (FBS) (Merck, Darmstadt, Germany), 100 units/mL of penicillin, and 100 μg/mL streptomycin. The virus was identified and its 50 % tissue culture infectious dose (TCID50) (PMID: 32458296) determined in 96-well plates containing 1 % FBS and confirmed by Rt-qPCR assays (artus^®^ Infl./H1 LC/RG RT-PCR kit; Qiagen, Germany) run with a Rotor-Gene Q Series 2.1.2-build 9 software (Qiagen N.V., Venlo, the Netherlands).

The virus stock containing 1×10^7^/mL TCID_50_ was diluted tenfold in cold, serum-free DMEM. A total of 4 mL medium containing the diluted virus was aerosolised in a sterile 50 mL Falcon tube, passed through the cartridge, and captured at the outlet in a 15 mL Falcon tube containing 4 mL of cold serum-free DMEM. Viral titre was determined by virus titration assays (19). All the experiments were repeated twice.

### Ozone measurement procedure

UV light can generate ozone, especially when the wavelength is below 240 nm, by breaking the bonds of oxygen molecules (20). Although the wavelength used in this study is 275 nm, we measured ozone to make sure that none were produced in the process. Ozone was measured at the cartridge outlet with the Indigo colour test, a passive visual technique based on colour change, using a Draeger ozone tube (Ozone 10/a, Drägerwerk AG & Co. KGaA, Lübeck, Germany).

## RESULTS AND DISCUSSION

**Figure 5:**
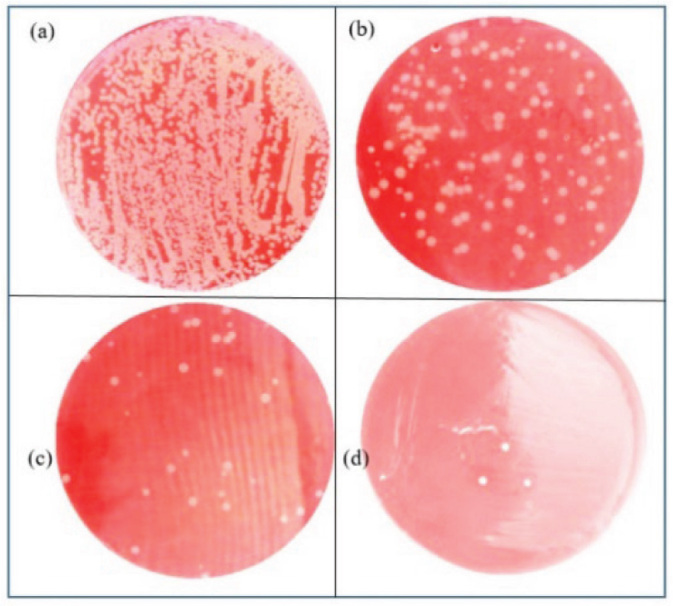
Efficiency against *S. aureus* with the three-layer cartridge and varying UVC light power, (a) no UVC light, (b) 50 mW, (c) 100 mW, and (d) 150 mW

**Figure 6:**
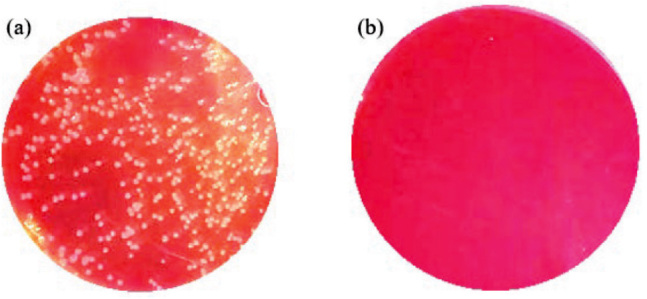
Efficiency against *S. aureus* with the one-layer cartridge at no UV light (a) and 75 mW UVC light power (b)

**Figure 7:**
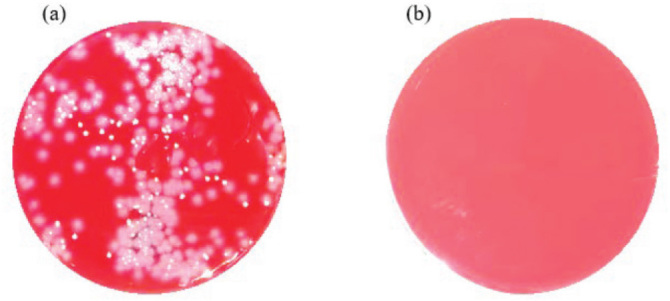
Efficiency against *P. aeruginosa* with the one-layer cartridge at no UV light (a) and 75 mW UVC light power (b)

Figures 5 to 7 show the efficiency of UVC LEDs at different output power against the bacteria, first with the three-layer (30-LED) cartridge (Figure 5) and then with the optimised one-layer (8-LED) cartridge (Figures 6 and 7) compared to control (no-LED) cartridge. The 150 mW UVC light power and the length of the three-layer cartridge air duct (around 200 cm combined) killed nearly all (99.9 %) of *S. aureus* (Figure 5d). At lower output power, it killed 87.8 %, (50 mW, 10-LED) and 93.5 % (100 mW, 20-LED) (Figure 5b and 5c, respectively).

**Figure 8:**
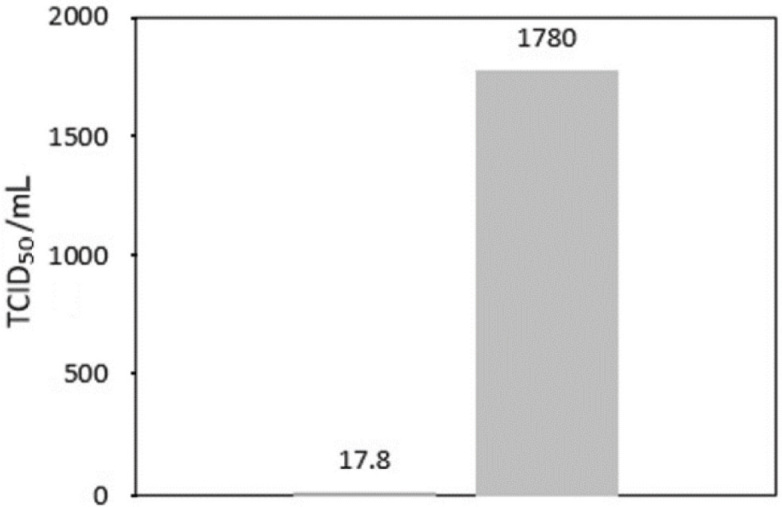
Comparison of viral titres (TCID_50_/mL) obtained with the active (75 mW) one-layer cartridge (left column) and control (no-LED) cartridge (right column)

The one-layer (8-LED) cartridge using the 75 mW UVC light output power against *S. aureus* and *P. aeruginosa* seems as efficient (Figures 6 and 7). Figure 8 shows that the one-layer cartridge was quite efficient against the influenza A/Puerto Rico/8/1934 virus, as the viral titre of the control cartridge was 100 times higher than that of the active cartridge emitting 75 mW of UVC LED light.

Ozone measurements showed no colour change on the indigo test, which clearly shows that ozone production during UVC radiation was negligible.

Even though they are encouraging, our findings have some limitations pertinent to a pilot (preliminary) study such as ours. Only further detailed research on a larger sample (which may include other pathogens).

Limitations aside, our face mask seems a promising solution for personal protection against germs: it is small, light, and easy to use. Moreover, its UVC cartridge system is efficient even without additional mechanical filtering. If necessary, mechanical filtering can easily be added to the mask. In addition, no UV light can leak from the device, being a closed system, and cause harm. Further design considerations should include blower-induced vibration, power consumption, and real-time monitoring of light intensity.
